# Minimally invasive brain injections for viral-mediated transgenesis: New tools for behavioral genetics in sticklebacks

**DOI:** 10.1371/journal.pone.0251653

**Published:** 2021-05-17

**Authors:** Noelle James, Alison Bell

**Affiliations:** 1 Neuroscience Program, University of Illinois at Urbana-Champaign, Urbana, Illinois, United States of America; 2 Department of Evolution, Ecology and Behavior, University of Illinois at Urbana, Urbana, Illinois, United States of America; 3 Carl R. Woese Institute for Genomic Biology, University of Illinois at Urbana-Champaign, Urbana, Illinois, United States of America; 4 Program in Ecology, Evolution and Conservation Biology, University of Illinois at Urbana-Champaign, Urbana, Illinois, United States of America; Chang Gung University, TAIWAN

## Abstract

Behavioral genetics in non-model organisms is currently gated by technological limitations. However, with the growing availability of genome editing and functional genomic tools, complex behavioral traits such as social behavior can now be explored in diverse organisms. Here we present a minimally invasive neurosurgical procedure for a classic behavioral, ecological and evolutionary system: threespine stickleback (*Gasterosteus aculeatus*). Direct brain injection enables viral-mediated transgenesis and pharmaceutical delivery which bypasses the blood-brain barrier. This method is flexible, fast, and amenable to statistically powerful within-subject experimental designs, making it well-suited for use in genetically diverse animals such as those collected from natural populations. Developing this minimally invasive neurosurgical protocol required 1) refining the anesthesia process, 2) building a custom surgical rig, and 3) determining the normal recovery pattern allowing us to clearly identify warning signs of failure to thrive. Our custom-built surgical rig (publicly available) and optimized anesthetization methods resulted in high (90%) survival rates and quick behavioral recovery. Using this method, we detected changes in aggression from the overexpression of either of two different genes, arginine vasopressin (*AVP*) and monoamine oxidase (*MAOA*), in outbred animals in less than one month. We successfully used multiple promoters to drive expression, allowing for tailored expression profiles through time. In addition, we demonstrate that widely available mammalian plasmids work with this method, lowering the barrier of entry to the technique. By using repeated measures of behavior on the same fish before and after transfection, we were able to drastically reduce the necessary sample size needed to detect significant changes in behavior, making this a viable approach for examining genetic mechanisms underlying complex social behaviors.

## Introduction

Complex behaviors have been repeatedly shown to be (reviewed in Dochtermann et al., [1]), yet establishing a causal relationship between genes and social behavior remains challenging. Partially, this difficulty arises from limitations of the primarily correlative methods for examining the interplay between genes and behavior (reviewed in Charney [2]) and partially due to small effect sizes [3, 4]. While quantitative trait locus (QTL) mapping, genome-wide linkage/association studies (GWAS) and modern sequencing technologies like RNA-Seq studies have nominated thousands of candidate genes important for natural behavior, it remains challenging to characterize the mechanisms and underlying neural circuitry. To fully characterize how a gene contributes to behavior, it is necessary to consider not just sequence differences, but also regulatory and epigenetic influences. Therefore, to demonstrate and fully characterize a causal relationship between a gene and behavior, it is crucial to have a method for manipulating gene expression at a specific time and location [5].

There is a growing toolbox for potentially manipulating gene function in non-traditional model organisms and it is important to select the right tools as each method has its own assumptions as well as necessary capabilities within the system [6]. Here, we focus on developing a minimally invasive neurosurgical method for direct brain injection for viral-mediated transgenesis in threespined stickleback (*Gasterosteus aculeatus*) fish, a foundational organism in ethology (reviewed in Huntingford & Ruiz-Gomez [7]). This procedure can deliver either pharmacological agents to test signaling pathways or transgenic elements to directly alter gene expression into the stickleback brain. Using direct brain injection, we examined the impact of candidate genes on territorial aggression via pharmacological manipulation and viral-mediated transgenesis.

The primary alternative to viral-mediated transgenesis is full germline transgenesis, typically accomplished through the microinjection of DNA directly into the nucleus of a fertilized ovum. Viral-mediated transgenesis is attractive in comparison for studying behavior, because manipulation specifically in the brains of adult animals avoids off-target, developmental, and compensatory effects. Moreover, viral-mediated delivery of constructs directly to the brain follows a timeline that is more conducive to behavioral studies in longer-lived animals. This approach has already proved essential in the functional testing of genes related to behavior [5, 8, 9] as well as in the dissection of neural circuits [10].

Viral-mediated transgenesis can be used to increase or decrease gene expression levels, using either a direct gene payload or a CRISPR cassette, respectively [11, 12]. The choice of promotor included in the viral payload allows researchers to tailor expression profiles and durations to their experimental needs. For example, pilot work for this study examined a short-term promotor, mCMV, that produced expression as early as 4 days post-injection, as well as a long-term promotor, hCMV, with expression lasting at least 5 weeks. Similarly, different viral vectors provide a choice of temporary episomal expression or, through retroviral vectors, permanent integration into the host genome. This method of transgenesis is fast and flexible and amenable to experimental designs in which the same individual animals are measured before and after gene expression is altered, allowing us to show a causal relationship. By using a repeated measures within-subject design, each animal acts as its own control, which statistically controls for variation between individuals. This is a major benefit when dealing with high inter-individual variation, such as when studying behavior or outbred animals collected from natural populations.

Threespine stickleback are already one of the best-studied animals for behavior and have a growing molecular toolkit including a fully sequenced genome [13]. Thus, sticklebacks are now gaining popularity in other fields including evolution, physiology, comparative genomics, and neuroscience [14–16]. They have been used in comparative cross-taxa studies looking for a conservation in the molecular underpinnings of social behavior with both emerging and classic model systems [17, 18], as well as in the evolution of behavior [7, 14, 19, 20]. Indeed, there are already hundreds of previously identified candidate genes for social behavior waiting to be characterized [21–27]. Sticklebacks enjoy well-established behavioral assays [28, 29] that are amenable to automation [15, 30].

There is a dearth of information on surgical methodology in small (3–4 cm) fish, despite their popularity as behavioral model systems, including zebrafish (*Danio rerio*) [31], medaka (*Oryzias latipes*) [32], and stickleback [7]. Anesthetization remains one of the most challenging aspects of surgery because proper anesthetizations timing must be determined on an individual basis. In modifying a zebrafish viral-mediated transgenesis procedure [33], we first needed to refine the anesthesia process [34, 35] to enable a longer and more precise surgery. Importantly, we introduce the use of an oral cannula providing oxygenation and maintenance anesthetic throughout the longer (10 minutes out-of-water) procedure. Water supplied by the cannula flows through the gills and has the secondary effect of keeping the clamping sponges and animal’s body surface wet. To improve the precision of brain injections, we designed and built a custom surgical rig as there are no commercially-available, water compatible, tiny stereotaxis tables. To maximize animal welfare, we additionally needed to identify clear warning signs of failure to recuperate by establishing a normal recovery pattern in stickleback similar to the work in koi by Harms et al., [36]. With this equipment and the ability to provide early intervention, survival rates rose to 90%.

To facilitate future behavioral work with this stereotactic brain surgery for stickleback, we tested methods to minimize post-surgical downtime such as supplemental oxygenation and verified prompt behavioral recovery via a simulated territorial intrusion immediately after physiological recovery. As the first test of this method in this species, we chose to focus on territorial aggression for three reasons: 1) it is a well-established, easy to score behavioral assay [15, 28, 37–39], 2) aggression is important for fitness [40, 41], and 3) there are good candidate genes for aggression based on studies in other vertebrates [18, 42, 43].

Here we employ this neurosurgical method to test the function of two conserved candidate genes related to aggression in stickleback: arginine-vasopressin and monoamine oxidase. Using viral-mediated transgenesis via direct brain injections, we tested if behavior level changes were detectable due to brain-wide overexpression of either *AVP* or *MAOA* in wild-caught male sticklebacks. To increase the accessibility of viral-mediated transgenesis in sticklebacks, a system with roots in ethology rather than genetics, we demonstrate the use of the technique for behavioral genetics by using widely available mammalian plasmids, rather than synthesizing or cloning out the stickleback gene sequences. To confirm the efficacy of pharmaceutical manipulations via brain injections, we compared brain and systemic injection of vasotocin.

Arginine-vasopressin (*AVP*) and its nonmammalian homolog arginine-vasotocin (*AVT*) are highly conserved [44] and pleiotropic [45] oligopeptides. Vasopressin (CYFQNCPRG) and vasotocin (CYIQNCPRG) are distinguished by only a single amino acid change between mammals (human) and teleosts (sticklebacks), and their respective V_1a_ receptors have similar specificity, signaling mechanisms, and amino acid sequences [46]. Both vasopressin and vasotocin were found to have similar physiological effects in rats [47]. Additionally, vasotocin signaling has been shown to influence aggression in various contexts in both fish and mammals (reviewed in Goodson [48]) and has been characterized throughout the social decision making network (SBDN) in the brain [49–52]. In fact, nonapeptide hormones (vasopressin/vasotocin, isotocin/mesotocin, and oxytocin) interact with sex steroids to influence behavior [46, 53], making them quintessential behavioral candidate genes.

In sticklebacks, vasotocin peaks during the start of the breeding season in both males and females [54]. Nesting male sticklebacks have an increase in vasotocin levels in their brains following a mirror (aggression) challenge [55]. The arginine-vasopressin-like (*avpl*) gene showed the greatest overexpression in dominant versus subordinate zebrafish [56], further supporting its role in aggressive behavior in teleosts. Vasotocin in adult teleosts is mainly located in the preoptic area (POA) of the hypothalamus [49, 57, 58], where it is an active regulator in the hypothalamic-pituitary-adrenal (HPA) axis [59]. Therefore, we hypothesized that supplemental expression of arginine vasopressin (*AVP*) within the social decision making network of the stickleback brain would increase aggression.

Exogenous vasotocin administration has been used in many teleosts and other vertebrates to alter behavior, both via intercranial and systemic injection [46], and has a dosage based response [60–62]. Additionally, given its role as an antidiuretic hormone [63], vasotocin produces a rapid physiological response of increased respiration, making its physiological effects quick and non-invasive to monitor. Vasotocin can pass through the blood-brain barrier [64, 65]. Therefore, unless brain trauma from intercranial injection rendered the technique unsuitable, we expected vasotocin to produce similar effects when administered either by intercranial or intraperitoneal (IP) injection.

Monoamine oxidase, our other candidate gene, has longstanding associations with psychopathologies, particularly aggression and anxiety [66], not only in model systems but also in humans [67]. Monoamine oxidase is an enzyme that cleaves and thereby inactivates several biogenic amines, including the neurotransmitters serotonin, norepinephrine, and dopamine [68]. These neurotransmitters are widely recognized to play critical roles in the regulation of cognition, mood, stress, and motivation [69]. Serotonin and dopamine levels have been suggested to follow an “inverted U” relationship with cognition, such that too little or too much activity both detrimentally affect performance [70, 71].

Teleosts have only one monoamine oxidase gene (*MAO*) as opposed to the two found in mammals (*MAOA* and *MAOB*). Stickleback *MAO* (ENSGACT00000012444.1) and mouse *MAOA* (NP_776101.3) have 68% conservation at the protein level. Despite the low level of conservation, the teleost monoamine oxidase gene is functionally comparable [68, 72].

Most prior examinations of the link between monoamine oxidase and aggression examine downregulation or knockout of *MAOA*, and the commensurate increase in serotonin activity. Mice with low or no *MAOA* activity showed increased fearfulness as juveniles and increased aggression in adult males, and increased risk of antisocial behavior [66, 68]. In teleosts, as in mice and humans, higher levels of serotonin at the synapse are associated with reduction of aggression [73]. In zebrafish, aggressive interactions lead to increased serotonergic activity in both winners and losers [73]. Administration of a conspecific alarm substance to zebrafish resulted in decreased MAO protein levels and increased aggression [74].

There is also preliminary evidence, however, that upregulation of *MAO* may also increase aggression. Many of the serotonin and dopamine pathway genes, including *MAO*, are upregulated in dominant fish [73], with overexpression of *MAO* observed in specifically in the hypothalamus [56]. In humans and mice, upregulation of *MAOA* is associated with increased anxiety [75, 76]. During the mating season, males solitarily guard their nesting territories [38]; increased anxiety could easily produce increased vigilance and irritability. Therefore, increased expression of *MAOA* was expected to increase aggression through serotonin turnover.

## Materials and methods

### Overview

In a ten-minute neurosurgical procedure, a suspension of foreign material (saline, viral construct, or pharmacological agent) was administered to the anterior diencephalon of the brain via transcranial injection. For the viral-mediated transgenesis portion of the experiment, bilateral injections delivered a total of 600 nL of replication-deficient Herpes Simplex Virus 1 (HSV-1) containing genetic payloads of either the *EYFP* fluorescent control or mammalian cDNA ORF clones of *AVP* or *MAOA*.

To facilitate comparison to previous studies, the experiment also included pharmacological treatments of exogenous vasotocin, Manning compound (a V_1_ receptor antagonist and anti-vasopressor), or saline. Vasotocin and saline were delivered both by the same transcranial protocol and by intraperitoneal injection as previously established in sticklebacks. Manning compound was delivered only by intraperitoneal injection.

The behavioral assay examined territorial aggression by recording the experimental individual’s response to an intruder confined to a glass flask, a well-established assay [28, 29] in sticklebacks. Baseline assays were taken at least 24 hours in advance of any manipulation. Experimental condition assays were taken at appropriate timepoints depending on the method of intervention: 30 minutes after IP injections, 2 hours after pharmacological brain injection, or at 14 and 16 days after viral-mediated injections. Respiration rate was also recorded immediately prior to each behavioral trial.

Finally, numerous pilot experiments refined the protocols used prior to the actual behavioral experiment. These examined, among other things, the choice of promotor to drive gene expression in viral-mediated transgenesis, location and depth of transcranial injection and their qualitative relationships to the area of cells transfected, the use of craniotomy as opposed to piercing the skull directly with the injection needle, the effect of needle gauge on survival and recovery rates, the time course for respiration rate to return to normal following transcranial injection, and the effect of supplemental oxygenation during surgical recovery on survival rate.

### Animals

Freshwater adult fish were collected in spring to summer in 2016 to 2018 from Putah Creek, CA. Additional F1 fish from crosses generated in 2015 and 2016 and reared in the lab were also used for optimizing the neurosurgical procedure. All fish were housed in the lab in 83 L (107x33x24 cm) group tanks with recirculated freshwater (5 ppm Instant Ocean Sea Salt). The room was maintained at 18°C on a 16:8 (L:D) “breeding” photoperiod from April to October and otherwise an 8:16 (L:D) “non-breeding” photoperiod. Fish were one to two years old at the time of their surgery. Males were identified by nuptial coloration (secondary sexual characteristics) and by sexing via PCR [77]. Individual males were weighed and measured (standard length from nose to caudal peduncle), overall averaging a length of 47.5 mm ± 2.66 (mean ± s.d.) and weight of 1.40 g ± 0.25 (mean ± s.d.). Then, they were moved to individual 9.5 L (32x21x19 cm) tanks lined with gravel and containing a synthetic plant. Each individual was allowed to acclimate, undisturbed for three days prior to any behavioral measurements. All fish only underwent one injection or surgery and were not reused.

### Ethics statement

All animal work was done in compliance with Institutional Animal Care and Use Committee protocol (IACUC #15077 and 18080) and Institutional Biosafety Committee protocol (IBC-4140) at the University of Illinois at Urbana-Champaign. During all simulated territorial intrusions, the resident and intruder were separated to prevent any injuries from occurring. Group housing was used to provide a normal, ecologically relevant, social environment prior to and following the experiment. All capture of fish was done under a Scientific Collecting permit (SC-3310) from California Fish and Game to the highest standards (IACUC and US law) by trained researchers with prior approval. Collections are kept to a minimum to avoid disrupting the native population. Fish receive daily maintenance of feeding and illness checks by lab members. Additionally, daily room and water checks are done by university Division of Animal Research technicians.

### Surgical rig

**Fig 1:**
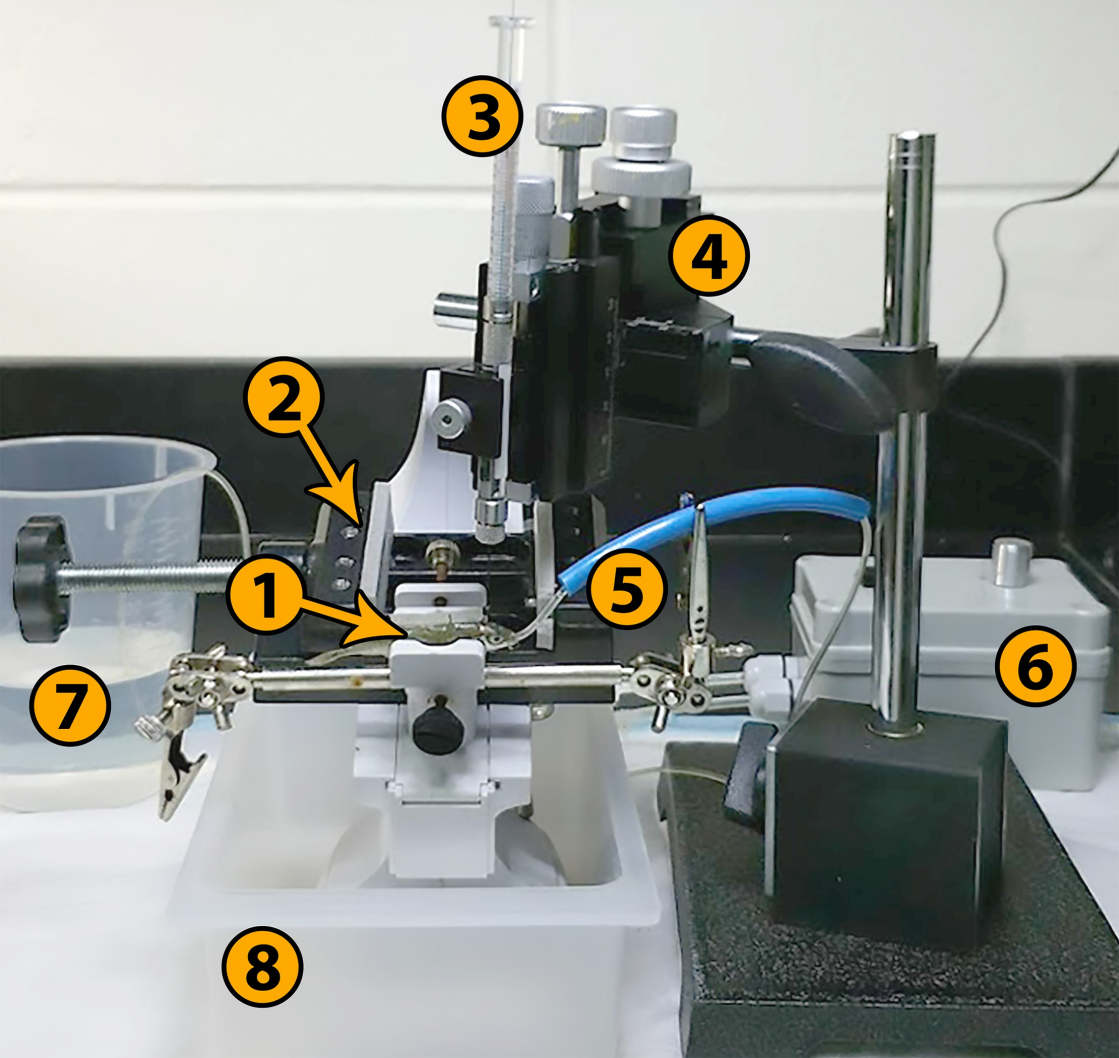
Custom-built surgical rig. 1. Threespine stickleback in padded. Clamp. 2. Alternative padded clamp for larger fish. 3. Neuros syringe, 5 μL. 4. Three-axis manipulator. 5. Oral cannula and guide tube. 6. Peristaltic cannula pump, 100 mL/min. 7. Pump source reservoir. 8. Drip tray.

For this procedure, we developed a custom-built surgical rig (Fig 1). To provide continuous oxygenation and anesthesia to the fish while out of water, a low pressure and flow rate cannula pump was necessary. The complete parts list along with assembly instructions are publicly available through the Open Science Framework (https://osf.io/sgpvm).

### Anesthesia

Prior to anesthetization, a pre-surgical baseline respiration rate was taken by counting opercular beats per 20 seconds. Initial anesthetization was done by immersion in 0.02% buffered MS-222 (Tricane-S, Western Chemical, Fisher) for no more than five minutes (188.4 sec ± 74.0, mean ± s.d.), until movement ceased and the fish was unresponsive. A properly sedated fish had 1) no tail movement, 2) decreased but regular respiration (opercula beating), 3) came to rest on the bottom of the soaking container, and 4) did not respond to touch nor move when removed from the bath.

Anesthetization time was not correlated with any physiological measure (S1 Fig). Fish were rinsed in freshwater (5ppm Instant Ocean Sea Salt) to remove any residual anesthetic then moved to the surgical rig. In the rig, an oral cannula supplied constant water flow with 0.01% MS-222 maintenance anesthetic over the gills for the duration of the surgical procedure (233s ± 79, mean ± s.d.). The speed of water delivery was adjusted to each fish to allow a steady low flow rate over the gills.

### Neurosurgical optimizations for direct brain injections

**Fig 2:**
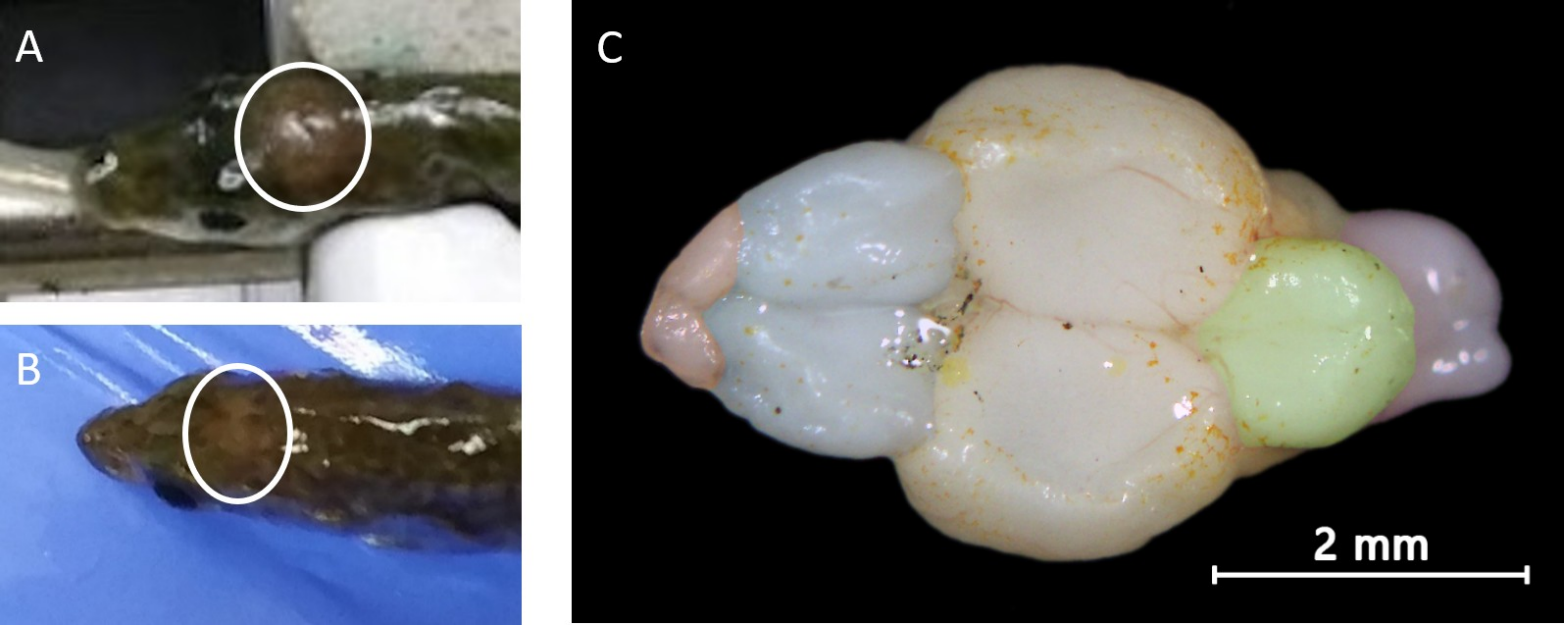
Dorsal visualization of the stickleback brain. A & B) Example visualizations of the stickleback brain through the skull. Circled is a lighter area corresponding to the diencephalon. C) The brain of a stickleback with olfactory bulbs (anterior, far left, colored red), telencephalon (left, colored blue), diencephalon and mesencephalon (center, natural color), cerebellum (right, colored green), and brain stem (posterior, far right, colored purple). Nodes of the social decision-making network are present in the telencephalon (amygdala, hippocampus, etc) and diencephalon (preoptic area (POA), hypothalamus, and periaqueductal gray/central gray (PAG)) as described in O’Connell and Hofmann [50].

Fish were gently clamped into the surgical rig behind the eyes, keeping the skull firmly in place. The stickleback diencephalon is visualizable through the skull (Fig 2), allowing injection sites to be selected with moderately high precision. In pilot studies we targeted either the telencephalon or the anterior diencephalon. Anecdotally, we saw better success rates in hitting the larger diencephalon rather than the small and more occluded telencephalon. Therefore, for the behavioral candidate gene work, all injections were targeted to the anterior diencephalon.

In each injection, a 33G (0.210 mm OD) needle attached to a 5 μL borosilicate syringe (Hamilton Neuros model 75, #65460–02, Reno, NV) was inserted transcranially through the thinnest portion of the skull posterior to the frontal bone [78], i.e. where the brain can be visualized (Fig 2). In pilot studies, we also examined the use of an insulin syringe (BD 328431, Franklin Lakes, NJ) with a 30G (0.337 mm OD) needle. The finer 33G needle had a hard stop set at 2.5mm. Each transcranial injection delivered a total of ~300 nL of liquid at one to three depths to moderate the area transfected, with one depth being the most restrictive. The depths of injection ranged from a maximum of 2.5mm beneath the surface of the head to as shallow as 0.3mm below the cranium’s surface, with ~0.7mm difference in heights for multiple depth injections. Bilateral transcranial injections delivered a total of ~600 nL. Any notable accumulation of blood was associated with poor outcome.

Following the procedure, fish were returned to their individual tanks and monitored continuously until clear respiration (opercular movement) was seen, typically within 30 seconds. In the case of shallow respiration, forced movement of fresh water over the gills was used to promote survival by manually “swimming” the fish in a submerged figure eight using only forward motion. Respiration rate and the fish’s position in the water column was recorded every 15 minutes for two hours following the injection. Additional checks were performed at three hours and one-day post-injection for all fish. Out of 183 total fish receiving brain injections, 19 did not survive this initial three-hour recovery period; nine did not survive anesthetization and ten were euthanized.

### Changes to direct brain injection procedure specific to viral-mediated transgenesis experiment

In order to accommodate animal biosafety level 2 (ABSL-2) restrictions from the use of HSV-1, fish used in viral-mediated transgenesis protocols were transferred into a new tank in the surgery room the morning of the injection. To maximize the probability of producing detectable behavioral changes, we aimed for broad expression throughout both hemispheres of the diencephalon (Fig 2C). Therefore, every fish received two bilateral transcranial injections to the anterior diencephalon of one of the viral constructs delivering a total of ~600 nL of construct across multiple depths from a 5 μL borosilicate syringe with 33G needle. After two days, fish were removed from the ABSL-2 surgical room to individual tanks.

### Pharmacological treatments

Exogenous [Arg8]-Vasotocin (Genscript RP10061, Piscataway, NJ) was administered either directly into the diencephalon of the brain via injection using the neurosurgical protocol described above or systemically via intraperitoneal (IP) injection, already a well-established method in sticklebacks, using a 30G (0.312 mm OD) insulin needle. Because behavioral response has been reported to differ in teleosts based on dosage [60, 61], a dose-response curve (0.5, 5, and 10 μg per gram body weight) was tested. Manning compound (Bachem H-5350.0001, VWR), a potent V₁ receptor antagonist (anti-vasopressor) was administered systemically via IP injection at a dosage of 3 μg per gram body weight. Both pharmacological agents were freshly diluted on the day of injection from a pre-suspended concentrated stock solution such that all IP injections delivered 10 μL per gram body weight. Behavioral assays were performed 48 hours prior to pharmacological manipulation for baseline measurements, and then at 30 minutes after IP injection or 2 hours after brain injection. Preliminary saline injections showed that 30 minutes was sufficient for both physiological and behavioral recovery from the IP injection procedure (full data: https://osf.io/v56zt).

### Viral-mediated transgenesis constructs

We elected to use Herpes Simplex 1 (HSV-1) as a viral vector as it was already shown to be effective in transfecting teleosts, where adeno-associated viruses (AAVs) have previously failed [33]. The modified virus is replication deficient, meaning it no longer carries the HSV-1 genome. Instead, it carries a genetic payload of DNA packaged as a plasmid, with a gene and promotor both of our choosing. This genetic construct is delivered into the animal cells through transfection by virions and then episomally expressed, with expression levels and duration determined by the choice of promotor used on the plasmid. As HSV-1 is not a retroviral vector, the plasmid remains in the cytoplasm and neither integrates into nor replicates with the genome. It is therefore transient, although it can persist for long periods of time in some cells [79]. Additionally, HSV-1 generally has low genotoxicity and no detectable shedding [80] reducing biohazard concerns. Finally, HSV-1 supports payload plasmids of larger size than AAVs or retroviral vectors such as lentivirus [8, 81].

**Fig 3:**
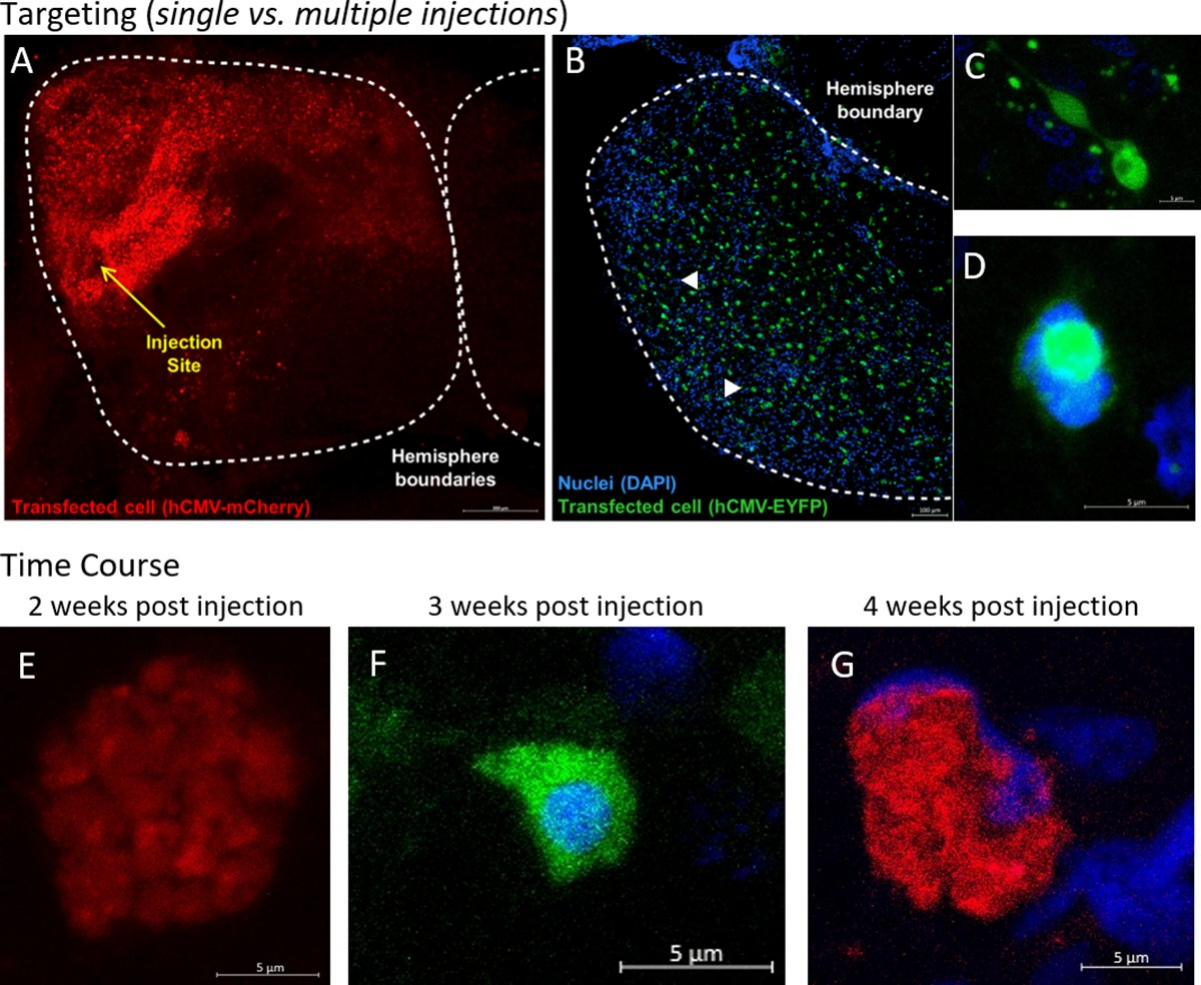
Fluorescent expression resulting from viral-mediated transgenesis. More refined targeting of brain regions is possible by limiting the number of injections. A) Single injection resulting in local expression, limited to a portion of one hemisphere of the anterior telencephalon. B) Broad expression throughout the entire left hemisphere of the diencephalon, typical for injections with delivery at multiple depths. No fluorescence was seen in any saline injected controls. C & D) Successful transfection of cells by the long term hCMV-EYFP construct in the lateral left diencephalon shown in B at three weeks after injection. E-G) Fluorescent expression profile over time for the hCMV promoter. No fluorescence was seen during the 10 days following injection. Weak but increasingly strong signal was seen between 10–12 days (not shown) following injections regardless of the fluorescent protein, mCherry (E & G) or EYFP (F). Peak expression was observed beginning around day 13 or 14 post injection. There was no qualitative difference in fluorescent intensity between two to four weeks, the maximum examined time point, following injection. Behavior was assessed at 14- and 16-days post injection.

Three promoters in replication deficient HSV-1 were piloted to drive gene expression based on work in zebrafish [33]–a long-term promoter (hCMV, *N* = 43, Fig 3) resulting in fluorescent signal 2–5 weeks after injection, a short-term promoter (mCMV, *N* = 10) with expression between 4 and 7 days post-injection, and a retrograde promoter (hEF1a, *N* = 7) which did not result in a detectable fluorescent signal. Promoters were tested for their ability to drive a fluorescent protein (EGFP, EYFP, GCaMP6f, or mCherry). The long-term promoter (hCMV) was selected as the most useful due to its longer window of effect and was used in the viral-mediated transgenesis experiment.

Mammalian cDNA ORF clones were used for *AVP* (human, HG17671-UT, NCBI Ref Seq: NM_000490.4, Sino Biological, Beijing, China) and *MAOA* (mouse, MG57436-U, NCBI Ref Seq: NM_173740.3, Sino Biological). These were cloned into the pDONR221 backbone (Epoch Life Science, Missouri City, TX) and then packaged (Gene Delivery Technology Core, Massachusetts General Hospital, Boston, MA) with an IRES-GFP backbone in replication deficient HSV-1 (proprietary, Rachael Neve). Stock hCMV-EYFP (RN12) was used for control injections. All males were randomly assigned to one of the three constructs. The final viral solutions were used undiluted except for the addition of a trace amount of pigment (brilliant blue FCF or tartrazine, i.e. FD&C Blue No. 1 and Yellow No. 5), ≤2% v/v during experiments, to allow the solution to be visualized against the gradations of the syringe.

### Behavioral assays

All behavioral data were gathered double-blind to treatment (saline, pharmacological agent or transfected gene). Respiration rate was determined prior to the territorial challenge by averaging two separate non-continuous counts of opercular beats per 20 seconds taken within a 5-minute period. This ensured that individual variations due to stress from the researcher’s activity were minimized. Territorial aggression was measured by recording the individual’s response to an intruder confined to a glass flask (derived from Wootton, 1971 [39]). The times to orient toward and to first bite at the intruder (TTO and TTB in our data files, respectively) were recorded, as well as the total number of bites, charges (lunges), and trips (approaches) during the five minutes following initial orientation (behaviors defined in Wootton, 1971 [39]). Intruders (*N* = 9) were 5–10% smaller conspecific males.

**Fig 4:**
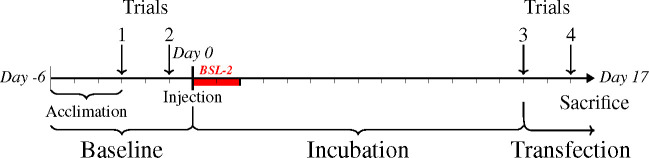
Experimental timeline with the injection of constructs on day 0. Fish were injected with a randomly assigned construct of either an aggression-related gene (AVP or MAOA) or a control fluorescent protein (EYFP). All trials were conducted double-blind to the transfected gene. Each trial had two respiration rate measurements followed by a territorial challenge.

During the viral-mediated transgenesis experiment, males’ respiration rate and behavioral response to a territorial challenge were recorded four times (Fig 4): twice before and twice after injection, respectively considered baseline and transfected. Each focal male except one was confronted by the same intruder during all four territorial challenges. In the exception, the initially paired intruder died between trials two and three and was replaced with a new male of the same length.

### Histology

Fish were sacrificed via decapitation with sharp scissors without anesthesia. Decapitation was used to cease brain activity as quickly as possible and because blood was also collected for supplemental hormone analysis (not shown) which is sensitive to anesthesia. Brains were immediately dissected out. Brains were mounted in glycerol medium on slides with four well iSpacers (SunJin Lab IS018, Hsinchu City, Taiwan). Imaging was performed on the same day as dissection on a Zeiss LSM 710 at the Core Facilities at the Carl R. Woese Institute for Genomic Biology. No fluorescence was seen in any saline injected controls.

### R statistical analysis and data availability

Boxplots are drawn with a heavy stroke at the median and whiskers of up to 1.5 IQR beyond the upper and lower quartiles. All data analysis was carried out in RStudio (v1.1.383) with R version 3.5.1. All scripts and data are publicly available on the Open Science Framework (https://osf.io/v56zt) as “Neurosurgical Protocol scripts.R” for the neurosurgical optimization, “AVP pharma treatment behavior.R” and as “Behavioral experiments scripts for release.R” for the viral-mediated transgenesis. Each individual was considered to be an experimental unit and no animals were excluded from analysis.

Survival rate differences for the neurosurgical optimization were calculated using the chi-squared function with continuity correction. A nonparametric-compatible repeated-measures ANOVA was done via the MANOVA.RM (v0.3.2) package with the ANOVA type statistic (ATS) reported because the assumption of sphericity could not be met for respiration rate over time (Mauchly tests for sphericity = 0.02, *p-value* = 1.65e-74). We report the ANOVA-Type Statistic (ATS) and the adjusted degrees of freedom, the latter of which are based on the number of treatment levels, number of observations, and the variance of ranks in each treatment [82]. For interaction effects, we report the recommended $F_{\left(\hat{f},\infty \right)}$ instead of $F_{\left(\hat{f},{\hat{f}}_{0}\right)}$ [83]. Post-hoc calculation by time point was done via Wilcoxon rank sum test with continuity correction and the rcompanion (v2.2.1) wilcoxonR function. P-values were then adjusted for false discovery rate (fdr method).

Repeatability is reported as ICC3,1 calculated using Desctool (v0.99.25) and confirmed with the nonparametric concordance package nopaco (v1.0.6). Significance was similar between the ICC and concordance tests. Spearman correlations were calculated using Hmisc (v4.1–1). Wilcoxon and Mann-Whitney tests were done with the base stats package and effect size was calculated with the rcompanion package (v2.2.1). Finally, sample size calculations utilized the WMWssp package (0.3.7) with the defaults of 0.05% for two-sided type I error rate and 0.8 power.

## Results

### Neurosurgical optimizations for direct brain injections

**Fig 5:**
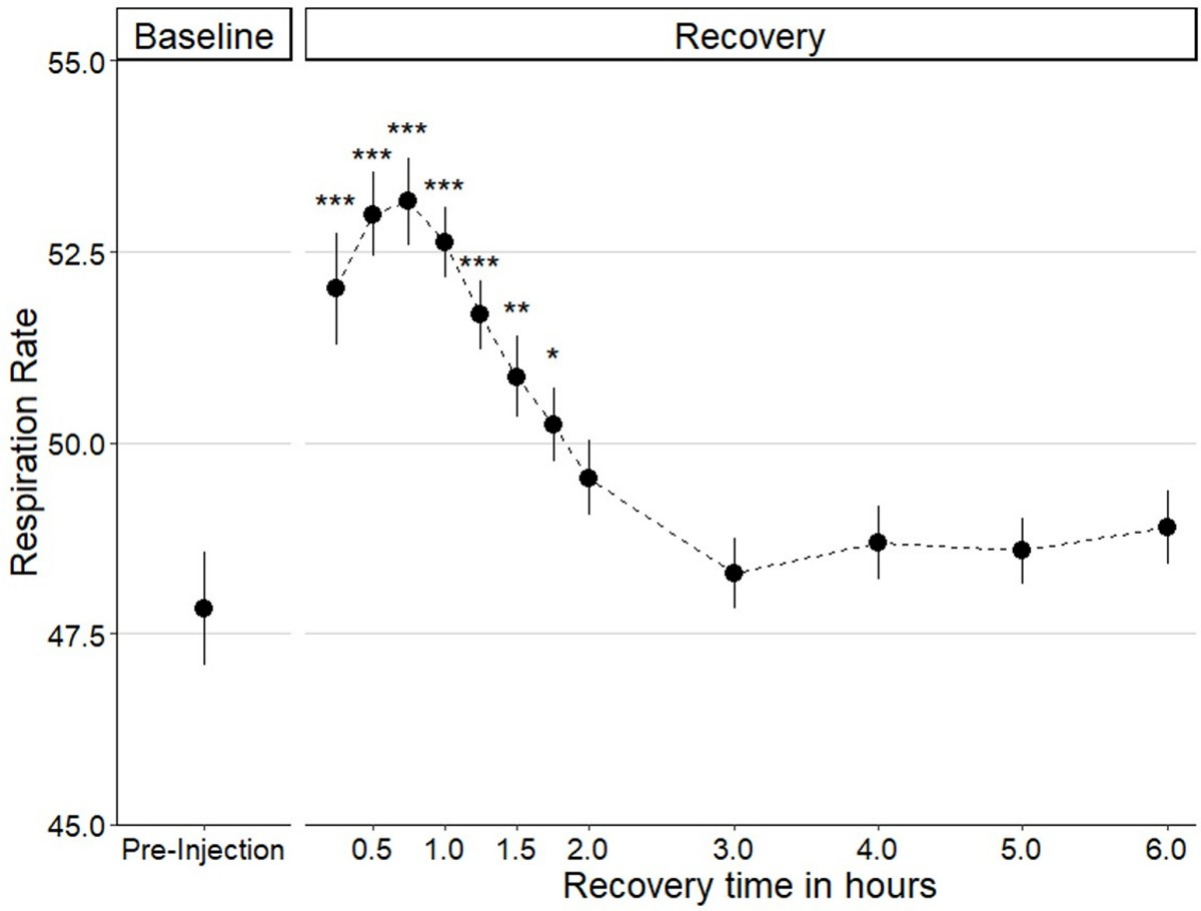
Average recovery curve of respiration rate following transcranial brain injections (*N* = 62). Respiration (opercular beats per 20 seconds) rate returned to baseline levels by two hours post-injection and remained stable following the surgery. Mean ± SE * p ≤ 0.05; ** p ≤ 0.01; *** p ≤ 0.001.

Direct transcranial injection with an unbeveled ultrafine needle proved simpler and more effective than other piloted techniques, including craniotomy. Fish generally returned to normal swimming and water column use within 15 minutes after removal of anesthesia. Initial piloting (*N* = 62) revealed respiration rate followed a typical pattern after the operation which we called a recovery curve (Fig 5). Respiration rate peaked about 30 minutes post-surgery and returned to baseline levels by two hours after the neurosurgical procedure.

**Fig 6:**
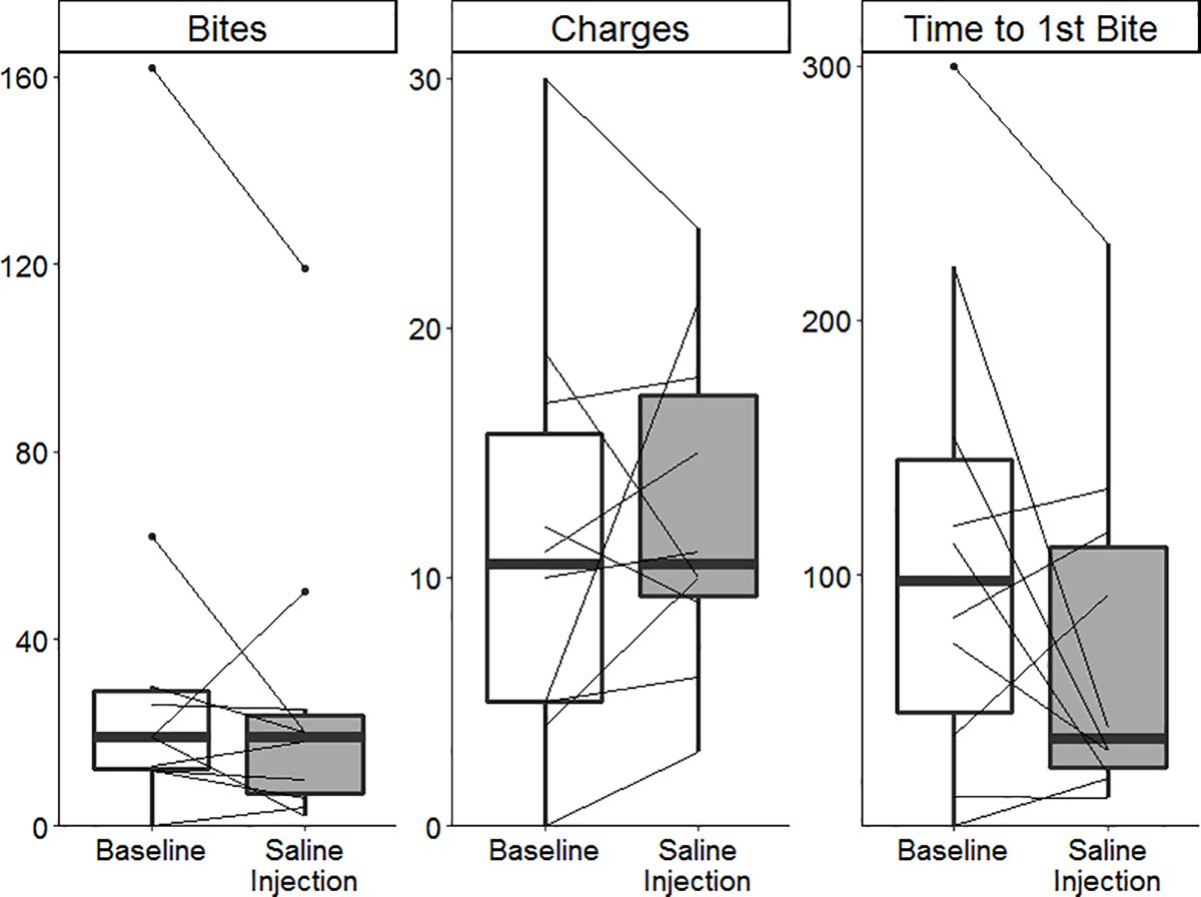
Aggressive behaviors in control fish one day prior to neurosurgery and after direct brain injection of saline (*N* = 10). Each line represents an individual showing their change in behavior following saline injection. Outliers beyond 1.5 IQR are shown as individual datapoints. No significant change in aggression was seen in any behavior.

There was no difference in territorial aggression two hours after brain injection in saline treated controls (*N* = 10), compared to the day before surgery in any aggressive behavior (bites: *Z* = -1.22, *p-value* = 0.21; charges: *Z* = -0.62, *p-value* = 0.54; time to first bite: *Z* = -1.22, *p-value* = 0.22, Fig 6). Full mating behavior occurred within three days for males, determined by nesting behavior and nine days for females, determined by the presence of eggs.

Needle diameter influenced survival rate (*χ*^*2*^ (1, *N*_30G_ = 43, *N*_33G_ = 183) = 23.9, *p-value* = 1.02e-6) with a 54% survival rate with the larger 30G needle compared to the 87% survival rate with the finer 33G needle. Additionally, the finer 33G needle resulted in consistently lower respiration rates (*ATS*_1, 2613_ = 6.54, *p-value* = 0.01) during the six-hour window following the neurosurgery than the larger diameter 30G needle. Supplemental oxygenation for up to two days following surgery did not improve survival (*χ*^*2*^ (1, *N*_Extra O2_ = 94, *N*_Normal_ = 89) = 0.02, *p-value* = 0.89) nor recovery (*ATS*_1, 7465_ = 0.94, *p-value* = 0.33, S3 Fig).

### Multiple transcranial injections do not alter survival or recovery

Broad expression required multiple transcranial injections–one into each hemisphere of the targeted brain region. Bilateral transcranial injections did not alter survival rates (*χ*^*2*^ (1, *N*_Unilateral_ = 64, *N*_Bilateral_ = 119) = 0.46, *p-value* = 0.50) compared to a unilateral injection. Overall, respiration rate during recovery was higher in fish with bilateral injections (*ATS*_1, 4874_ = 11.61, *p-value* = 0.001) in the three hours following the surgery. However, this comparison was largely confounded by year of capture, with the fish caught in 2018 all receiving two transcranial injections and showing poor health in general. When analyzing only years (2017 & 2019) that received both unilateral and bilateral injections, allowing for direct comparison, there is no difference in respiration rate during recovery between unilateral and bilateral injections (*ATS*_1, 312_ = 0.92, *p-value* = 0.34). This suggests that the differences in recovery among years overwhelmed any effect that multiple injections might have had.

### Injection material does not alter survival or recovery

Fish had comparable survival rates regardless of the injected materials (*χ*^*2*^ (3, *N*_*HSV-1*_ = 113, *N*_*Pharma*_ = 22, *N*_*Saline*_ = 48) = 2.30, *p-value* = 0.32). After the surgical technique was refined, the procedure’s survival rate was approximately 90%. Of the 113 fish injected with one of three constructs utilizing replication deficient HSV-1 for transfection, 101 survived. The fish injected with pharmaceutical agents fared similarly, with 20 of 22 fish surviving. Control fish injected with saline fared least well as they were used to initially pilot and refine the surgical technique; they counted 39 survivors among 48 fish, an 81% survival rate.

The time for recovery of fish injected with replication deficient HSV-1 did not differ from that of saline injected controls (*ATS*_3.7, ∞_ = 1.83, *p-value* = 0.12). Additionally, the choice of promoter (hCMV, mCMV, or hEF1a) did not alter survival rate (*χ*^*2*^ (2) = 1.38, *p-value* = 0.50), nor was there a main effect of promoter (*ATS*_2, 143_ = 0.33, *p-value* = 0.70) on the recovery curve. Finally, the specific gene being expressed had no effect (*χ*^*2*^ (3) = 2.16, p = 0.54) on survival rates relative to saline injected controls. The recovery rate was also unaffected by the gene expressed (*ATS*_2, 267_ = 1.19, *p-value* = 0.31).

### Vasotocin pharmacological treatment

**Fig 7:**
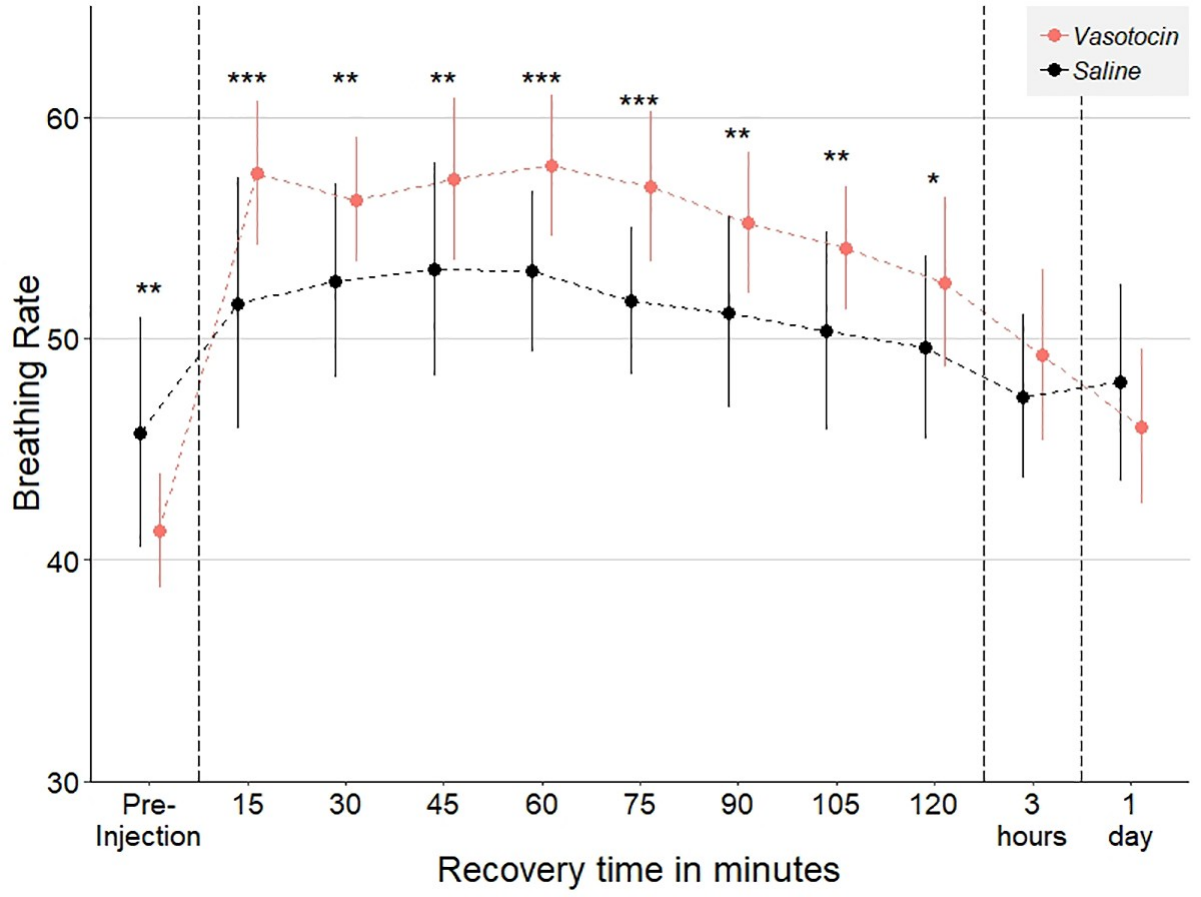
Differences in respiration rate following injection between brain injection of exogenous vasotocin (*N* = 15) and saline injected controls (*N* = 39). Fish injected with vasotocin had an elevated respiration rate compared to saline injected controls for more than two hours following injection. Mean ± SE * p ≤ 0.05; ** p ≤ 0.01; *** p ≤ 0.001.

Exogenous vasotocin injection into the brain increased respiration rate rapidly relative to saline injected controls (*ATS*_1, 599_ = 8.74, *p-value* = 0.003, Fig 7). This effect began within 15 minutes and persisted for more than two hours post-injection. The most pronounced difference in respiration rate was between 0.75- to 1.5-hour post-injection. Intraperitoneal injection of exogenous vasotocin also resulted in a rapid increase in respiration rate (S3 Fig). Behaviorally, brain and IP injections of exogenous vasotocin produced parallel results (S1 Table), in which only the highest dosage (10 μg per gram body weight) altered the number of charges directed at the intruder.

### Behavioral repeatability and intercorrelations

Repeatability was analyzed across the two baseline and two transfected trials. Charges, bites, and time to first bite were consistently repeatable, i.e. at both baseline and following transfection but not necessarily between baseline and transfection (S2 Table). Aggression measures were generally equally repeatable compared to the physiological measure of respiration rate. Total number of bites and charges were strongly correlated (*r* = 0.69) in the control group (*N* = 16) and following transfection of either *AVP* (*r* = 0.83, *N* = 18) or *MAOA* (*r* = 0.75, *N* = 20). Time to first bite was negatively correlated with total number of bites and charges as well–i.e. fish that bit sooner also attacked more overall. Given its high repeatability and correlation with other behaviors, we focus on charges as a measure of aggressive behavior in subsequent analyses.

### Increased aggression from transfection of MAOA or AVP but not in controls

**Fig 8:**
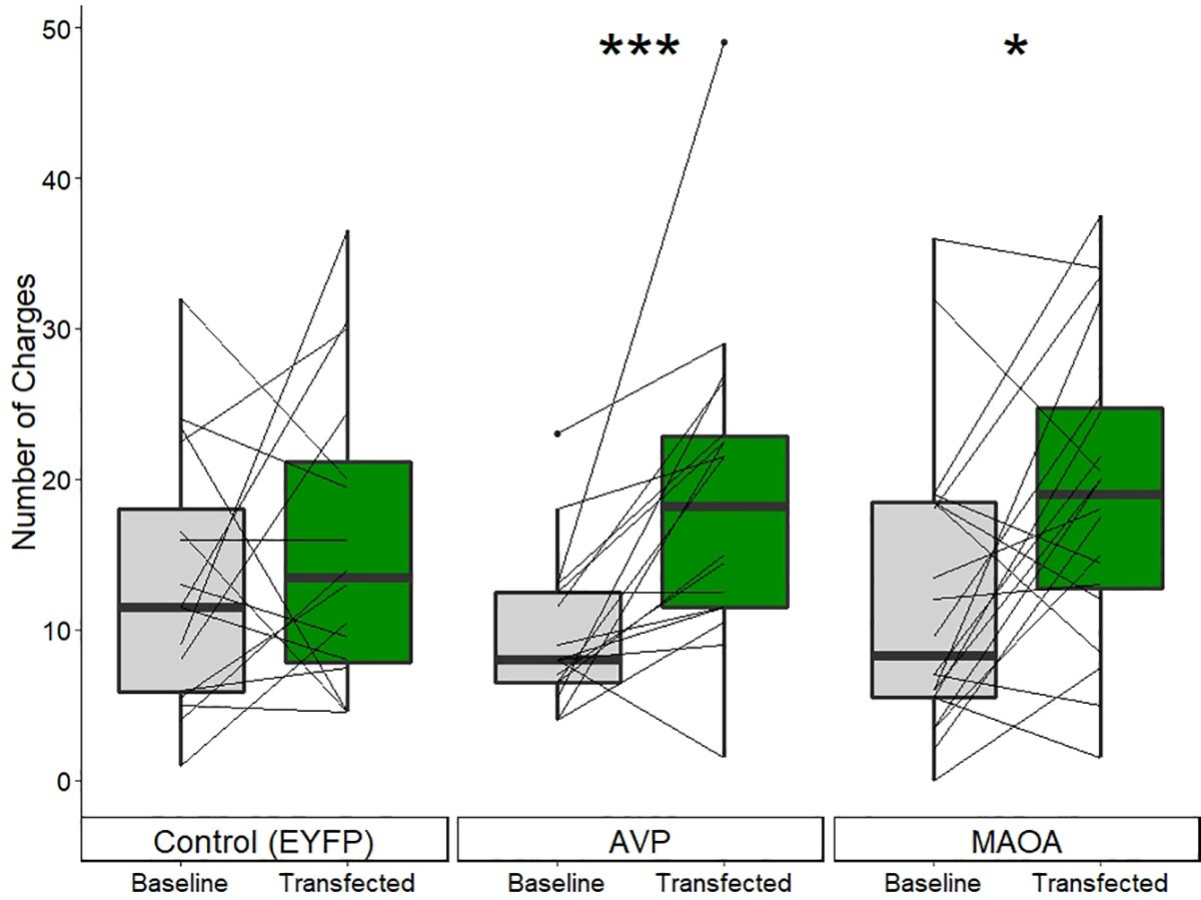
Number of charges (averaged across the two trials) before and after transfection for each construct. Each line represents an individual showing their change in behavior following transfection of the gene of interest. Transfection with AVP resulted in a substantial and consistent increase in the number of charges; note that only one individual exhibited decreased charging behavior. Transfection with MAOA resulted in an increase of large effect size in charges, although there was more variation in individual response. * p ≤ 0.05; ** p ≤ 0.01; *** p ≤ 0.001.

Aggressive behavior (charges, Fig 8) increased in fish transfected with either *AVP* (*N* = 18) or *MAOA* (*N* = 20) compared to the baseline measurements in those fish, i.e. in within-individual, repeated measures comparisons. Injection of the control construct (*EYFP*, *N* = 16) did not significantly alter the number of charges (paired Wilcoxon signed rank test: *Z* = -0.01, *p-value* = 0.50) relative to baseline. Due to the high inter-individual variation (σ^2^_*EYFP*_ = 103, σ^2^_*AVP*_ = 113, σ^2^_*MAOA*_ = 100), there was no significant difference between either *AVP-* or *MAOA-*injected fish compared to *EYFP*-injected controls in charges following transfection (Mann-Whitney test: *AVP*-*EYFP*: *Z* = -0.41, *p-value* = 0.34, *MAOA*-*EYFP*: *Z* = -0.7, *p-value* = 0.48), i.e. in comparisons between groups. No increase in aggression was seen in the saline injected controls neurosurgical optimization experiments (Fig 6). Thus, only injection with either of two aggression-related candidate genes resulted in altered behavior and only specifically in charges (S4 Fig).

*AVP* had a large effect on the number of charges (paired Wilcoxon signed rank test: *rs* = 0.79, *Z* = -3.07, *p-value* = 0.001) with 16 of the 18 individuals increasing the average number of charges compared to their baseline. In magnitude, this represented an almost 100% increase in average number of charges, from 9.7 (s.d. = 5.1) at baseline to 18.8 (s.d. = 10.6) following transfection.

Transfection with *MAOA* also caused a large increase in the average number of charges (paired Wilcoxon signed rank test: *rs* = 0.53, *Z* = -2.10, *p-value* = 0.018) relative to baseline. However, the effect of *MAOA* was less drastic than that of *AVP* and had more variation in individual response (Fig 8), with 13 of 20 individuals increasing their average number of charges. Despite this, *MAOA* still resulted in an approximately 50% increase from 12.1 (s.d. = 9.7) charges at baseline to 19.1 (s.d. = 10.0) following transfection.

### *MAOA* decreased respiration rate

**Fig 9:**
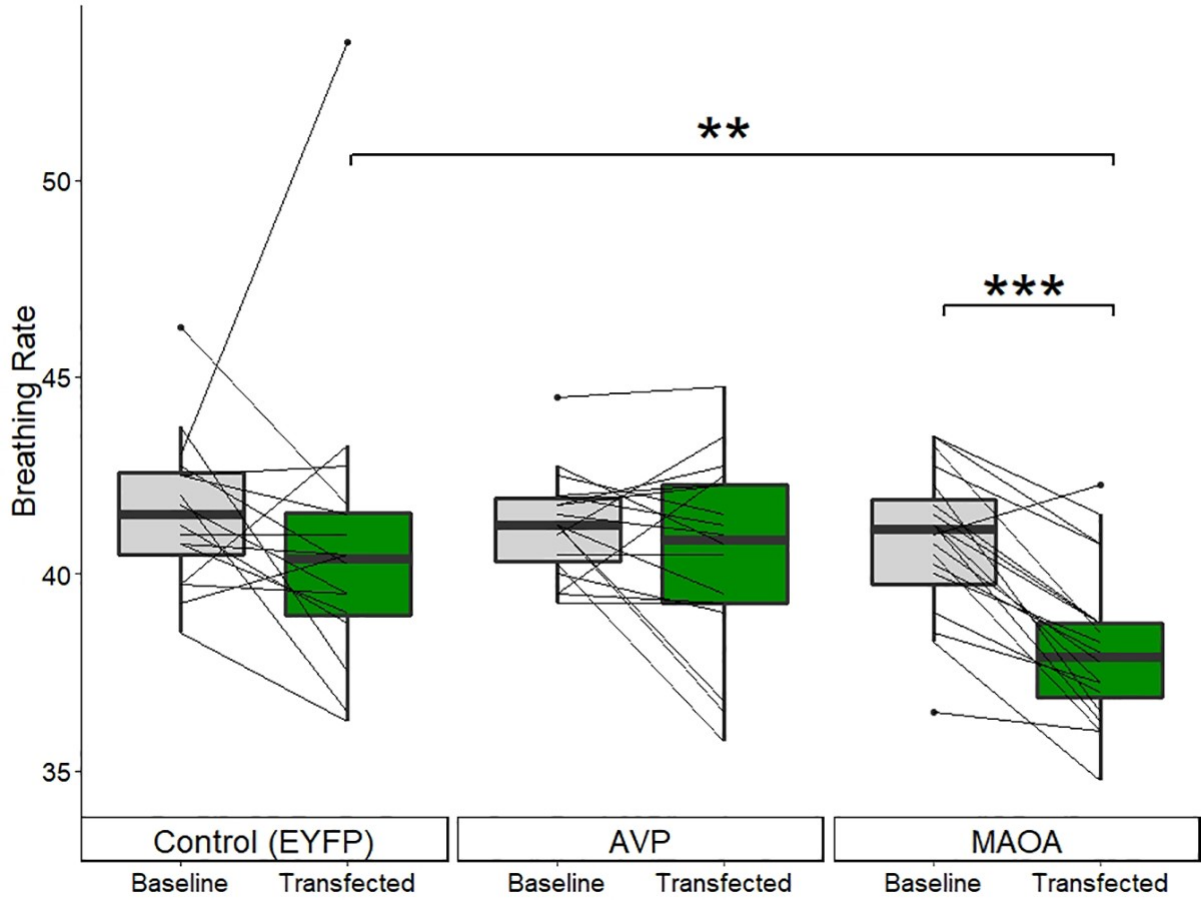
Respiration rate (opercular beats per 20 seconds) for the three constructs. Each line represents an individual showing their respiration rate (averaged across the two trials) before and after transfection. Only MAOA altered respiration rate; a drastic decrease compared to both baseline (within-subject comparison, N = 20) and to the control group (N = 16). * p ≤ 0.05; ** p ≤ 0.01; *** p ≤ 0.001.

Only the *MAOA* construct altered respiration rate (Fig 9). Compared to baseline, *MAOA* strongly and significantly (*N* = 20, *rs* = 0.85, *Z* = -3.62, *p-value* = 0.0001) lowered respiration rate, with 19 of the 20 individuals experiencing a decrease in resting respiration rate. They dropped from an average of 40.8 (s.d. = 1.9) to 38.1 (s.d. = 2.0) breaths per 20 seconds. Additionally, when comparing the respiration rates between the fish transfected with *MAOA* and the *EYFP* controls (*N* = 16, *mean* = 40.8, s.d. = 3.9), the decrease was still significant, though reduced to a moderate effect size (Mann-Whitney test: *rs* = 0.44, *Z* = -2.65, *p-value* = 0.009). There was not significant change in respiration rate compared to baseline due to either the control *EYFP* (*Z* = -1.11, *p-value* = 0.13) or *AVP* (*Z* = -0.92, *p-value* = 0.18) constructs.

## Discussion

### Neurosurgical optimizations for minimally invasive direct brain injections

We present a new method for direct injection of transgenic or pharmaceutical material into the brains of the small teleost fish threespined stickleback. Developing a minimally invasive neurosurgical protocol required 1) refining the anesthesia process, 2) building a custom surgical rig, and 3) determining the normal recovery pattern allowing us to clearly identify warning signs of failure to thrive. Our surgical rig and optimized anesthetization methods [34, 35] resulted in high (90%) survival rates and quick behavioral recovery. Mating behavior also recovered promptly: males completed nests at three days post-surgery, and females were gravid at nine days–suggesting almost no delay in the egg development time [84] after losing any ripe eggs to clamping during surgery.

Establishing a typical recovery curve (Fig 5) allowed us to identify post-surgical warning signs of failure to thrive. Behavioral manifestations of discomfort or problems included listing (>45° off central axis), assuming a nose-up position, and loss of positional control (twirling). The presence of any of these markers for greater than an hour suggested a poor prognosis and thus we recommend euthanasia. Survival to 24 hours indicated a successful procedure, as 23 out of 24 fish injected with the larger 30G needle and 159 of 160 fish injected with the smaller 33G needle survived to one week. Thus, this minimally invasive neurosurgical method is quite reliable.

### Pharmacological manipulation: Bypassing the blood-brain barrier

Exogenous vasotocin administered directly to the brain produced physiological and behavioral responses mirrored in fish receiving vasotocin through IP injections (Fig 7, S3 Fig and S1 Table). These pharmacological results were similar to those seen in other fish [56, 61, 85]. This indicates that brain injection is now a feasible delivery route for drugs that do not pass through the blood brain barrier [86] in sticklebacks. Ultimately, similar changes in respiration and aggression suggest that the recovery period from the brain injection does not mask even rapid onset pharmaceutical effects, like those due to vasopressin [87].

In this experiment, similar physiological effects were produced throughout the dose-response curve (S3 Fig). In conjunction, although behavioral effects were produced only at the highest dosage, they were similar for both routes of administration (S1 Table). These consistencies suggest that the pharmacological manipulation did in fact work.

### Viral mediated transgenesis: Genetic underpinnings of territorial aggression

To demonstrate the practicality of viral-mediated transgenesis to examine candidate genes’ contribution to behavior, we looked for behavioral changes from two aggression related candidate genes. It is well established that both vasopressin (*AVP*) and monoamine oxidase (*MAOA*) influence aggression [48]. Using a ubiquitous promoter resulted in altered territorial aggression specifically for fish receiving either one of the candidate genes related to aggression (Fig 8), but not for a fluorescent protein (Fig 8) or following saline brain injection (Fig 6). The effect following transfection of *AVP* was consistent, with 16 of the 18 fish experiencing an increase in aggression, one remaining constant and only one decreasing aggression.

The effect of *AVP* transfection was of stronger magnitude (*rs =* 0.79) than the effect of pharmacological manipulation of vasotocin (*r*_*Brain*_ *=* 0.66, *r*_*IP*_ *=* 0.67). This is likely due to the broad expression throughout the diencephalon. It is highly likely that our transgenic procedure resulted in ectopic expression as vasotocin is typically only produced in the preoptic area (POA) and ventral hypothalamus (VH) [31, 57]. Maximal expression was desired to increase the likelihood of transfection and of producing behavior changes through supraphysiological levels of vasotocin signaling. It will be fruitful for future studies to examine the consequences of altered expression in key brain areas and cell types, as discussed below.

Multiple pieces of evidence indicate that we achieved successful transgenesis, in addition to the consistent changes in both behavioral (Fig 8) and physiological (Fig 9) phenotypes following transfection. The most visible, literally, is the presence of novel fluorescent proteins (Fig 3). Additionally, transcripts specifically of the injected mammalian *MAOA* homolog remained detectable through qPCR for up to 4 weeks after transfection (results not shown).

Our finding that transfection of *MAOA* increased aggression is consistent with a decrease in serotonin, which is enzymatically cleaved by monoamine oxidase. Indeed, the clear and unambiguous decrease in respiration rate we observed (Fig 9) is strong evidence of *MAOA* functioning as expected physiologically. Respiration rate correlates positively with serotonin and norepinephrine concentration [88, 89]; monoamine oxidase enzymatically lowers levels of both neurotransmitters. Additionally, trout monoamine oxidase has been found to be equivalently effective to human monoamine oxidase in metabolizing 5-HT and PEA [68], making it unlikely that the increase in aggression is an off-target effect of using mammalian *MAOA*. Further characterization of anxiety levels following transfection, pharmacological rescue [90], and quantification of the downstream neurotransmitters remain as potential avenues to a better mechanistic understanding of this result.

We found that construct selection is not limited to native genes; widely available mammalian plasmids successfully altered behavior. Therefore, this method is accessible to a broad array of users [91], a feature especially important in a system with roots in ethology. Furthermore, this application of viral-mediated transfection enables within-subject experimental designs, reducing sample sizes required for the same statistical power and making behavioral experiments viable, as detailed below. Finally, rapid behavioral recovery makes viral-mediated transgenesis a viable technique for direct manipulation of candidate genes.

### Future applications of viral-mediated transgenesis in stickleback

While sticklebacks are a non-traditional genetic model system, they are one of the best studied behavioral systems, with well described intra-specific variation in aggression, antipredator behavior, and parental care [7, 14, 92]. Previous studies have identified hundreds of genes that are differentially expressed in the brain in response to a social interaction [21–27]. However, most of these studies are correlative, and thus the direction of the causal relationship–much less the mechanisms by which changes in gene expression underlie behavior–are still not clear. This method will allow future studies to rigorously test how these genes contribute to behaviors, from detailed mechanistic analyses at the protein level to studies of individual behavior at the organismal level.

A more detailed, cross-population study of vasotocin in stickleback would present an ideal opportunity to investigate the evolutionary constraints or trade-offs between behavior and physiology for pleiotropic genes. In addition to being associated with behavior, vasotocin plays a key role in osmoregulation via the AVP V2 receptors. Vasotocin anatomy and aggression differed in concordantly with salinity and osmoregulation challenges in pupfish [93]. A more nuanced examination is possible in stickleback as there are numerous freshwater and several anadromous populations that are independently evolved from ancestral-like marine population. In addition, population differences in aggression [37, 94, 95] and osmoregulation [96] have already been documented in sticklebacks. However, an examination of the integration of these two evolutionary concerns has not yet been undertaken, despite a relationship between aggression and kidney size having already been discovered in stickleback bred for extremes of territorial aggression [97]. This makes stickleback uniquely suited system to address the relationship of physiological ecology, anatomy, and social behavior [48]. Other possible physiological signaling pathways amendable to genome editing in sticklebacks have also been proposed [13, 98–101].

### Behavioral response to vasopressin/vasotocin

In every case of vasotocin signaling manipulation that we tested–pharmacological IP inhibition (Manning compound) or supplementation, exogenous brain injection, and transfection–number of charges at the intruder was the main responding aggressive behavior (Fig 8 and S2 Table). Charges as the main responding behavior matches the effects of vasotocin seen in pupfishes [85]. This is of interest as charging marks voluntary initiation of aggression, while biting is an escalation based off intruder response [28]. However, depending on the type of manipulation we saw opposing effects on the number of charges–pharmacological supplementation of exogenous vasotocin resulted in a decrease while transfection with *AVP* resulted an increase. As previously discussed, there is reason to hold high confidence in both pharmacological treatments and the transfection method, despite the varying directions of effect.

These techniques have drastically different timings and durations of the increase in vasotocin, potentially explaining the differing directions of behavioral changes. The pharmacological manipulation involved a single dose and a behavioral assay after two hours, while the transgenesis experiment involved increasing exposure throughout a two-week transfection incubation period, with two behavioral assays over three days (Fig 4). Vasopressin has an extremely short half-life of less than a minute [87], making pharmacological manipulation rapid but ephemeral. In contrast, transfection is much longer lasting, allowing for long-term stabilization of the HPA axis and commensurately altered behavioral response.

Additionally, vasotocin signaling is mainly constrained by receptor type and location [48, 49, 58] further emphasizing the potential of long-term homeostasis to influence the behavioral outcome. To mimic this with pharmacological manipulation, future work should use an implanted cannula in the brain to deliver repeated low doses of exogenous vasotocin. This would allow a more direct comparison between treatment methods clarifying if the opposing effects of pharmacological manipulation and transgenesis are due to homeostatic balancing from long-term exposure or a potential side effect from an immune response to the injection of small molecules.

### Viral-mediated transgenesis allows statistically powerful repeated measures design

Complex phenotypes that emerge at the whole organism level, such as behaviors like aggression, are difficult to assay due to their subtleties and time-intensive screening. Social behaviors are influenced by many genes of small effect [3, 4] and social psychology generally has smaller effect sizes (*r*) relative to other psychological sub-disciplines [102]. Indeed, the neuroscience, psychiatry, psychology, and behavioral ecology fields are plagued with reports of overestimates of effect sizes [102–105]. Additionally, behavior in natural populations tends to have high inter-individual variation, further reducing statistical power [106]. For sticklebacks, who have a generation time of approximately one year, the traditional approach of breeding a stable transgenic line is not always practical. Here, by using within-subject design, we successfully examined two behaviorally relevant genes for effects on aggression in wild-caught fish.

By using repeated measures on the same fish before and after transfection, we were able to drastically reduce the necessary sample size needed to detect significant changes in behavior (Table 1). In this study we found large effect sizes for both behavior and respiration rate, a typical physiological measure. However, variation following transfection with *MAOA* was about 25 times larger for charging behavior (*σ*^2^ *=* 99.9) compared to respiration rate (*σ*^2^ *=* 3.9). A between group comparison would have required an impractical sample size of as many as 300 fish to detect the difference in charges, even though these genes have a large magnitude (*rs* > 0.5) of effect on behavior. However, by using these methods we were able to reduce the sample size down to merely 20 fish, a far more manageable number. Thus, viral-mediated transgenesis enables the study of genetic effects on natural behavior in wild-caught animals, because it makes possible a repeated measures design comparing within the same individuals, increasing sensitivity.

**Table 1:** Benefit of within-subject experimental design on sample size for behavioral genetic studies.

	Behavior *(Charges)*		Physiology *(Respiration)*
	*AVP*	*MAOA*	*MAOA*
Necessary sample size (between groups, vs. control)	277	187	35
Actual sample size	18	20	20
Effect size (*rs*)	0.79	0.53	0.85
Variance (σ^2^, post-expression)	113	99.9	3.9

Necessary sample sizes assume a statistical power of 0.8 and are based on the effect sizes observed in this study–i.e. 8 out of 10 experiments using samples this large will detect the difference. Viral-mediated transgenesis makes possible a repeated measures design comparing within the same individuals, increasing sensitivity. Note that AVP and MAOA are known to have a large effect on behavior, so this should be viewed a minimum for future candidate genes being screened for behavioral phenotypes.

## Conclusions

Viral-mediated transgenesis is a method to alter a gene’s expression in a specific location or during a controlled timeframe. Refinements to both the stereotactic procedure and the design of constructs promise improvements in the specificity of targeted brain areas and cell types, allowing manipulations of gene expression with great precision. We successfully used multiple ubiquitous promoters to drive expression, tailoring expression profiles through time. Localizing expression to specific brain areas is a priority for future work but restricting injection amounts and locations can lead to transfection failure. Therefore, we intend to prioritize cell- and region-specific targeting with alternate promoters (see Ingusci et al., 2019 [12]). There is also tremendous potential for this method to be used in combination with cell type specific promoters that, for example, target astrocytes (*GFAP*), glutamatergic (*vGLUT*), GABAergic (*GAD*), dopaminergic (*TH*) or prolactin (*PRL*) neurons.

When combined with pharmacological manipulation, DREDDs (designer receptors exclusively activated by designer drugs, reviewed in Roth, 2016 [107]) can target neural signaling with extremely precise timing. This method is already being used to identify neural circuits via chemical silencing or activation of receptors including those that are serotoninergic [12]. Finally, viral-mediated transgenesis also lays the groundwork for optogenetics, although there are still engineering challenges to design light, tether-free setups that do not interfere with complex behaviors in fish that weigh less than 2 grams.

Here we present a minimally invasive neurosurgical procedure for sticklebacks that enables viral-mediated transgenesis in the brain as well as pharmaceutical delivery directly to the brain. This method for viral-mediated transgenesis allows for a more direct examination of the genetic mechanisms underlying behavior in wild-caught animals from natural populations. It is flexible, fast, and allows us to compare individual behavior before and after transgenesis, maximizing statistical power. It further enhances the growing molecular toolkit in threespine stickleback, a classic ethological system. By using a system with extensively optimized behavioral assays, we had good sensitivity for different effects on aggression, as only the initiation of aggression (charges) was altered and not the escalation of aggressive interactions (bites). Overall, our experimental results show that viral-mediated transgenesis is a promising method for testing the function of candidate genes in this system. This approach has already proved essential in the functional testing of genes related to behavior [5, 8, 9] and in the dissection of neural circuits [10] in other organisms.

## Supporting information

S1 Fig Spearman correlations between time to anesthetization (*N* = 148), time for neurosurgical procedure (*N* = 124), respiration rate prior to surgery (*N* = 153), weight (*N* = 157) and length (*N* = 87). Anesthetization time was not correlated with any other measure. Larger fish had slower respiration rates and it took longer to perform the surgery on heavier fish, in large part due to increased care in clamping. Numerical values and color both represent the strength of the correlation with crossed out boxes indicating non-significance (*P* > 0.05).
(TIF)

S2 Fig Respiration rate (opercular beats per 20s) following injection either with (*N* = 83) or without (*N* = 77) supplemental oxygenation for one day following the neurosurgical procedure. Supplemental oxygenation did not significantly improve recovery rates. Mean ± SE.
(TIF)

S3 Fig Differences in respiration rate following IP injection of different pharmaceutical agents. Fish injected with vasotocin at low to moderate dosage had elevated respiration rates compared to saline injected controls at both 10 and 30m post injection, paralleling the pattern seen during recovery of brain injection. The highest dosage of AVP (10 ^μg^/_gbw_) only resulted in a significant elevation in respiration rates at 30m post injection compared to saline injected controls. There was no significant difference between Manning compound and saline injected controls in respiration rates at any time point. Mean ± SE * *p* ≤ 0.05; ** *p* ≤ 0.01; *** *p* ≤ 0.001.
(TIF)

S4 Fig Breathing rate and repeatable behaviors across all trials & constructs. Breathing rate decreased significantly following transfection of only MAOA. Charges increased following AVP or MAOA transfection but not in control EYFP fish. Graph presents medians with interquartile range bars.
(TIF)

S1 Table Within-subject comparison of territorial aggression following pharmaceutical manipulation of vasotocin signaling compared to baseline. Brain and IP injection results were similar with only the highest dosage altering behavior. No group (including the Manning treatment) significantly differed from saline-injected controls. * *p* ≤ 0.05; ** *p* ≤ 0.01; *** *p* ≤ 0.001.
(DOCX)

S2 Table Repeatability of territorial aggression behaviors and respiration rate across the two trials at baseline and after transfection using two-way mixed, single score ICC (type 3,1) for all fish (*N* = 54). *EYFP* is the control, *AVP* and *MAOA* are genes of interest.
(DOCX)
