# Supplementary figures and images for: Minimally invasive brain injections for viral-mediated transgenesis: New tools for behavioral genetics in sticklebacks
Source: PLoS One. 2021 May 17;16(5):e0251653. doi: 10.1371/journal.pone.0251653 (PMC8128275; doi:10.1371/journal.pone.0251653)

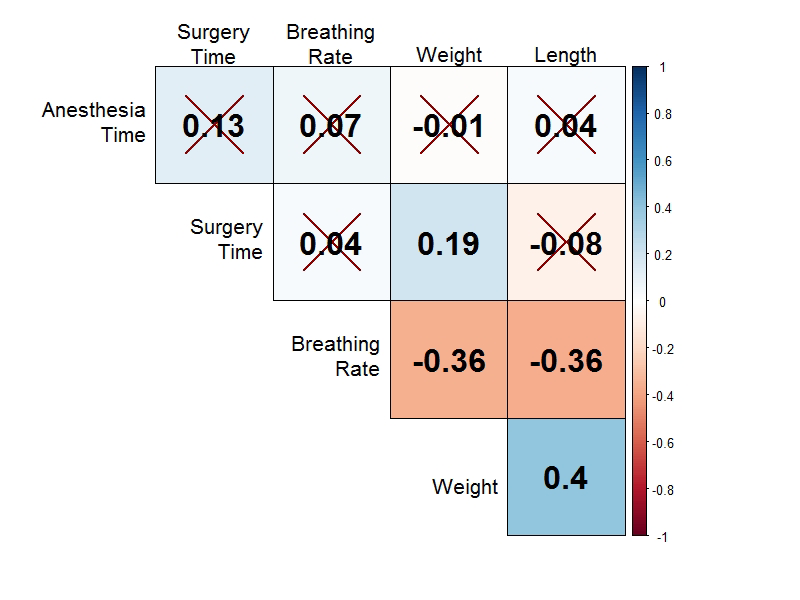

Supplement: S1 Fig — Anesthetization time was not correlated with any other measure. Larger fish had slower respiration rates and it took longer to perform the surgery on heavier fish, in large part due to increased care in clamping. Numerical values and color both represent the strength of the correlation with crossed out boxes indicating non-significance (P > 0.05). (TIF) [file pone.0251653.s001.tif]

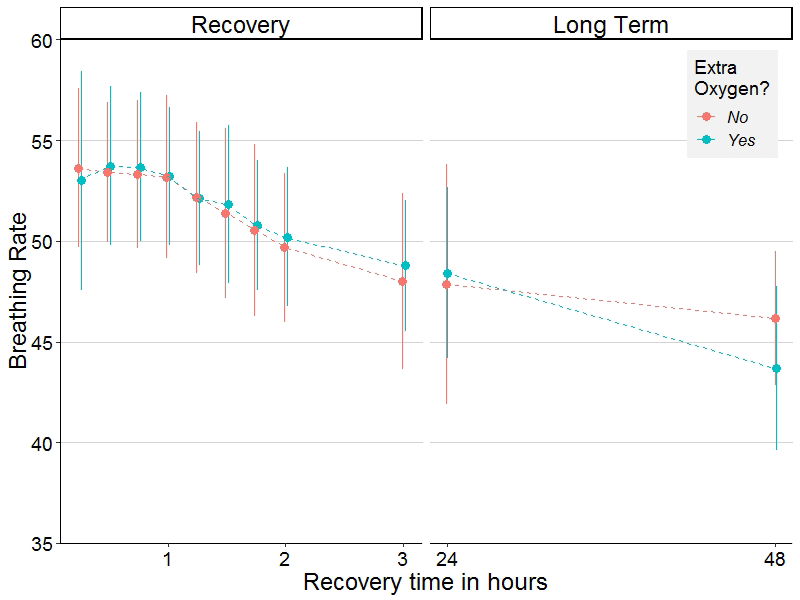

Supplement: S2 Fig — Supplemental oxygenation did not significantly improve recovery rates. Mean ± SE. (TIF) [file pone.0251653.s002.tif]

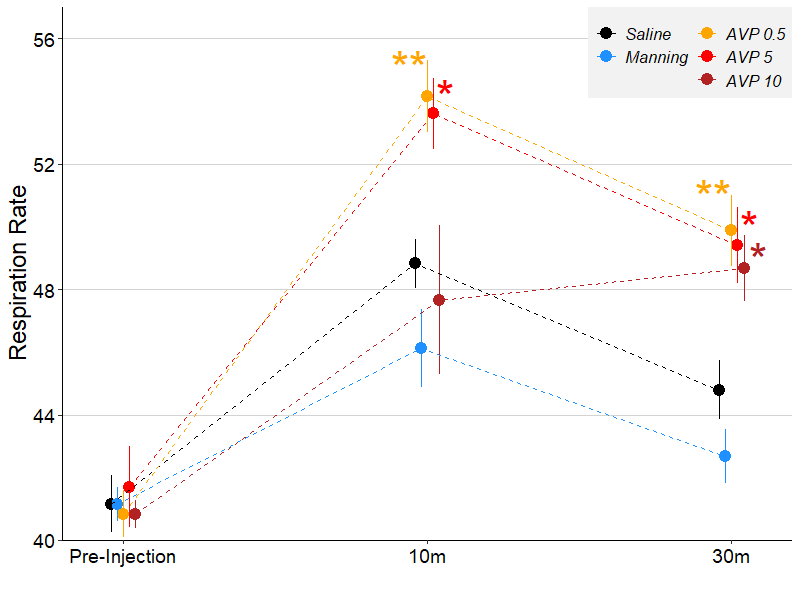

Supplement: S3 Fig — Fish injected with vasotocin at low to moderate dosage had elevated respiration rates compared to saline injected controls at both 10 and 30m post injection, paralleling the pattern seen during recovery of brain injection. The highest dosage of AVP (10 μg/gbw) only resulted in a significant elevation in respiration rates at 30m post injection compared to saline injected controls. There was no significant difference between Manning compound and saline injected controls in respiration rates at any time point. Mean ± SE * p ≤ 0.05; ** p ≤ 0.01; *** p ≤ 0.001. (TIF) [file pone.0251653.s003.tif]

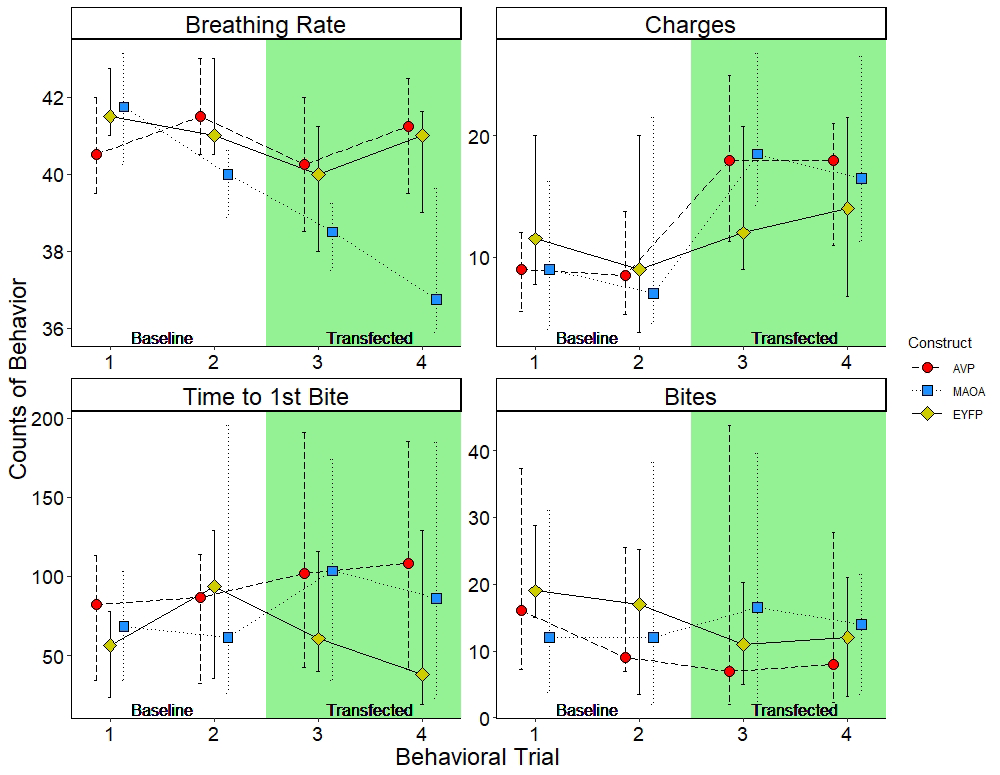

Supplement: S4 Fig — Breathing rate decreased significantly following transfection of only MAOA. Charges increased following AVP or MAOA transfection but not in control EYFP fish. Graph presents medians with interquartile range bars. (TIF) [file pone.0251653.s004.tif]
