# Supplementary material for: Minimally invasive brain injections for viral-mediated transgenesis: New tools for behavioral genetics in sticklebacks
Source: PLoS One. 2021 May 17;16(5):e0251653. doi: 10.1371/journal.pone.0251653 (PMC8128275; doi:10.1371/journal.pone.0251653)
Supplement: S1 Table — Brain and IP injection results were similar with only the highest dosage altering behavior. No group (including the Manning treatment) significantly differed from saline-injected controls. * p ≤ 0.05; ** p ≤ 0.01; *** p ≤ 0.001. (DOCX) [file pone.0251653.s005.docx]

|  | N | Bites | | | | Charges | | | |  |
| --- | --- | --- | --- | --- | --- | --- | --- | --- | --- | --- |
|  |  | Change | Z | Effect size (r) | P | Change | Z | Effect size (r) | P | |
| AVP (Brain, 0.5 ^µg^/_gbw_) | 3 | -29.3 | -1.3 | 0.93 | 0.18 | -2.0 | -0.9 | 0.82 | 0.35 | |
| AVP (IP, 0.5 ^µg^/_gbw_) | 16 | -10.0 | -1.6 | 0.41 | 0.11 | 1.8 | -1.1 | -0.29 | 0.25 | |
| AVP (Brain, 1 ^µg^/_gbw_) | 2 | -16.5 | -0.9 | 0.95 | 0.37 | -24.5 | -0.9 | 0.95 | 0.37 | |
| AVP (IP, 5 ^µg^/_gbw_) | 5 | -2.4 | -1.4 | 0.66 | 0.18 | -6.3 | -0.8 | 0.43 | 0.41 | |
| AVP (Brain, 10 ^µg^/_gbw_) | 10 | -17.8 | -1.5 | 0.52 | 0.12 | -8.2 | -2.0 | 0.66 | 0.04 * | |
| AVP (IP, 10 ^µg^/_gbw_) | 9 | -2.4 | -0.3 | 0.12 | 0.80 | -6.3 | -2.0 | 0.67 | 0.05 * | |
| Manning (IP) | 16 | -32.4 | -2.6 | 0.67 | 0.008 ** | -5.8 | -2.5 | 0.64 | 0.01 * | |
| Saline (Brain) | 10 | -8.1 | -1.2 | 0.40 | 0.22 | 1.4 | -0.6 | -0.21 | 0.54 | |
| Saline (IP) | 19 | 6.9 | -0.7 | 0.17 | 0.46 | -1.9 | -1.2 | 0.27 | 0.23 | |

**S1 Table. Within-subject comparison of territorial aggression following pharmaceutical manipulation of vasotocin signaling compared to baseline.** Brain and IP injection results were similar with only the highest dosage altering behavior. No group (including the Manning treatment) significantly differed from saline-injected controls. * *p* ≤ 0.05; ** *p* ≤ 0.01; *** *p* ≤ 0.001.
