# Supplementary material for: Minimally invasive brain injections for viral-mediated transgenesis: New tools for behavioral genetics in sticklebacks
Source: PLoS One. 2021 May 17;16(5):e0251653. doi: 10.1371/journal.pone.0251653 (PMC8128275; doi:10.1371/journal.pone.0251653)
Supplement: S2 Table — EYFP is the control, AVP and MAOA are genes of interest. (DOCX) [file pone.0251653.s006.docx]

| **Measure** | **Construct** | **Baseline** | | | **Transfected** | | | **Repeatable at both timepoints** |
| --- | --- | --- | --- | --- | --- | --- | --- | --- |
|  |  | **ICC** | **95% CI** | **p-value** | **ICC** | **95% CI** | **p-value** |  |
| Respiration Rate | *EYFP* | 0.49 | 0.01, 0.78 | 0.02 | 0.91 | 0.76, 0.97 | 2.51E-07 | Yes |
|  | *AVP* | 0.38 | -0.09, 0.71 | 0.05 | 0.73 | 0.42, 0.89 | 1.76E-04 | Yes |
|  | *MAOA* | 0.53 | 0.12, 0.78 | 0.007 | 0.35 | -0.09, 0.68 | 0.06 |  |
| Time to Orient | *EYFP* | 0 | -0.48, 0.48 | 0.50 | 0.13 | -0.37, 0.58 | 0.30 |  |
|  | *AVP* | 0 | -0.46, 0.46 | 0.50 | 0 | -0.46, 0.46 | 0.50 |  |
|  | *MAOA* | 0.04 | -0.40, 0.47 | 0.43 | 0.21 | -0.24, 0.59 | 0.18 |  |
| Time to 1st Bite | *EYFP* | 0.77 | 0.47, 0.91 | 1.32E-04 | 0.47 | -0.01, 0.78 | 0.03 | Yes |
|  | *AVP* | 0.51 | 0.07, 0.78 | 0.01 | 0.28 | -0.20, 0.65 | 0.12 |  |
|  | *MAOA* | 0.48 | 0.06, 0.75 | 0.01 | 0.74 | 0.45, 0.89 | 6.96E-05 | Yes |
| Bites | *EYFP* | 0.88 | 0.68, 0.95 | 2.0E-06 | 0.78 | 0.47, 0.92 | 1.17E-04 | Yes |
|  | *AVP* | 0.45 | 0, 0.75 | 0.03 | 0.73 | 0.42, 0.89 | 1.69E-04 | Yes |
|  | *MAOA* | 0.62 | 0.25, 0.83 | 0.001 | 0.59 | 0.21, 0.82 | 0.002 | Yes |
| Charges | *EYFP* | 0.64 | 0.23, 0.86 | 0.003 | 0.69 | 0.31, 0.88 | 0.001 | Yes |
|  | *AVP* | 0.55 | 0.12, 0.80 | 0.008 | 0.54 | 0.11, 0.80 | 0.01 | Yes |
|  | *MAOA* | 0.39 | -0.06, 0.70 | 0.04 | 0.83 | 0.62, 0.93 | 1.61E-06 | Yes |
| Trips | *EYFP* | 0.75 | 0.41, 0.90 | 2.82E-04 | 0.32 | -0.19, 0.70 | 0.10 |  |
|  | *AVP* | 0.39 | -0.08, 0.72 | 0.05 | 0.19 | -0.29, 0.59 | 0.22 |  |
|  | *MAOA* | 0.61 | 0.24, 0.82 | 0.002 | 0.66 | 0.31, 0.85 | 6.18E-04 | Yes |

**S2 Table.** **Repeatability of territorial aggression behaviors and respiration rate across the two trials at baseline and after transfection using two-way mixed, single score ICC (type 3,1) for all fish (*N* = 54).** *EYFP* is the control, *AVP* and *MAOA* are genes of interest.
